# Diet‐Related Differences Predominate Over Breed‐Associated Differences in Hepatic Lipid Metabolism, Antioxidant Vitamins and Lipid Quality Indices Among Beef Cattle

**DOI:** 10.1111/jpn.70102

**Published:** 2026-08-01

**Authors:** Laíza de Kássia Mendes da Conceição, José de Brito Lourenço‐Júnior, Jamile Andréa Rodrigues da Silva, Leonel António Joaquim, Mariana Jucá Moraes, Thomaz Cyro Guimarães de Carvalho Rodrigues, Joana Inês Ferreira, Ana Maria Ramos Fontes, Maria Pinheiro Spínola, Érica Coutinho David, Antônio Vinicius Correa Barbosa, Bruno Cabral Soares, José António Mestre Prates

**Affiliations:** ^1^ Graduate Program in Animal Science—PPGCAN (Federal University of Pará—UFPA, Brazilian Agricultural Research Corporation—Embrapa Federal Rural University of the Amazon—UFRA) Castanhal Brazil; ^2^ Federal Rural University of the Amazon (UFRA) Belém Brazil; ^3^ CIISA—Interdisciplinary Research Centre in Animal Health Faculty of Veterinary Medicine, University of Lisbon Lisbon Portugal; ^4^ Faculdade de Medicina Veterinária Av. Universidade Técnica Lisbon Portugal; ^5^ Associate Laboratory for Animal and Veterinary Science (AL4AnimalS) Faculty of Veterinary Medicine, University of Lisbon Lisbon Portugal; ^6^ Institute of Agricultural Research of Mozambique (IIAM) Estação Zootécnica de Angónia Angónia Moçambique; ^7^ Federal University of Roraima (UFRR) Boa Vista Brazil; ^8^ Beef Cattle Nutrition Center—CINPEC Paragominas Brazil; ^9^ Federal Rural University of the Amazon (UFRA) Paragominas Brazil

**Keywords:** antioxidant vitamins, beef cattle, fatty acids, lipid metabolism, liver, nutritional quality

## Abstract

Understanding how nutritional management and genetic background influence hepatic metabolism is important for improving the nutritional quality of bovine products in tropical production systems. This study evaluated breed‐associated differences under a common dietary regimen and diet‐related differences within the Nelore breed with respect to hepatic cholesterol, lipid‐soluble antioxidant vitamins, fatty acid composition and lipid quality indices. Thirty‐six intact male cattle were allocated to three experimental groups: Nelore fed a silage–concentrate diet, crossbred (½ Nelore × ½ Angus) fed the same diet, and Nelore fed a high‐grain diet. Hepatic cholesterol and antioxidant vitamins were determined by high‐performance liquid chromatography, whereas fatty acid composition was analysed by gas chromatography with flame ionisation detection. No significant differences were observed in hepatic cholesterol concentration for either the breed‐associated or diet‐related contrasts (*p* > 0.05). Breed‐associated differences were detected for β‐carotene, β‐tocopherol, γ‐tocopherol and docosahexaenoic acid (22:6*n‐*3) (*p* < 0.05). Diet‐related differences significantly affected β‐carotene, tocopherols, tocotrienols, several individual fatty acids and lipid quality indices, including the *n‐*6*/n‐*3 ratio and hypocholesterolaemic and hypercholesterolaemic fatty acid indices (*p* < 0.05). Principal component analysis showed clear separation of animals by dietary treatment, with β‐carotene, α‐tocopherol and tocotrienols contributing strongly to sample discrimination. Overall, diet‐related differences within the Nelore breed were associated with significant changes in a broader range of hepatic lipid and antioxidant variables, whereas breed‐associated differences under a common dietary regimen were observed for a more limited set of variables. These findings highlight the importance of nutritional management in modulating hepatic composition and support the use of liver biomarkers to assess metabolic responses in beef cattle.

## 1 Introduction

Intensive beef cattle production systems have expanded in response to the increasing global demand for animal protein and for products with enhanced nutritional and functional quality (Mazarin and Meneguelli 2025; Ponnampalam et al. 2024a). In this context, nutritional management plays a central role in modulating animal metabolism and tissue composition, particularly in tropical production systems, in which feeding costs represent the largest proportion of operational expenses (Serviço Nacional de Aprendizagem Rural SENAR 2018; Mazarin and Meneguelli 2025). In parallel, Espinales et al. (2024) and Ponnampalam et al. (2024a) highlighted the growing consumer interest in foods associated with reduced cardiovascular risk and increased intake of antioxidant bioactive compounds.

In ruminant physiology, the liver functions as a metabolically dynamic organ essential for regulating lipid and protein metabolism (Bionaz et al. 2020). Costa et al. (2014) reported that, although de novo fatty acid synthesis in cattle occurs predominantly in adipose tissue, the liver remains fundamental for the uptake of non‐esterified fatty acids (NEFA), cholesterol synthesis and lipoprotein metabolism. In addition, Makhutova et al. (2023) and Costa et al. (2014) demonstrated that hepatic tissue exhibits a lipid profile distinct from that observed in muscle and adipose tissue, functioning as an important reservoir of long‐chain *n‐*3 polyunsaturated fatty acids (LC‐PUFA), including eicosapentaenoic acid (EPA) and docosahexaenoic acid (DHA). Lobato et al. (2024) and Li et al. (2014) also described bovine liver as one of the most nutrient‐dense edible tissues, representing an important source of lipid‐soluble vitamins, carotenoids and essential minerals.

Alfaia et al. (2009) and De la Fuente et al. 2009) demonstrated that dietary composition and production system are the principal determinants of fatty acid composition and antioxidant status in ruminant tissues. Xu et al. (2015) observed that high‐concentrate diets markedly modify hepatic fatty acid metabolism, whereas Ponnampalam et al. (2024b) reported that forage‐based systems favour *n‐*3 polyunsaturated fatty acid deposition and promote more favourable *n‐*6:*n‐*3 ratios for human consumption. Schulz et al. (2025) identified greater hepatic β‐carotene concentrations in animals with increased pasture availability, whereas Lobato et al. (2024) observed higher hepatic concentrations of antioxidant compounds under specific feeding systems, reinforcing the direct influence of diet on hepatic metabolic responses and antioxidant status.

Genetic background also influences lipid metabolism and nutrient deposition patterns in cattle. Serviço Nacional de Aprendizagem Rural SENAR (2018) described differences between taurine and zebu cattle regarding nutrient utilisation efficiency and fat deposition, whereas Mukiibi et al. (2018) identified differential expression of hepatic genes associated with lipid metabolism among genetic groups. Costa et al. (2014) and Zachwieja et al. (2025) demonstrated that breed may particularly influence the deposition of specific long‐chain polyunsaturated fatty acids, such as DHA. Furthermore, Costa et al. (2014) identified significant interactions between breed and dietary energy level, indicating that animals with different genetic backgrounds respond differently to similar feeding strategies.

Despite the recognised metabolic importance of bovine liver, Lobato et al. (2024) and Rodrigues et al. (2023) highlighted that studies simultaneously evaluating hepatic cholesterol, lipid‐soluble antioxidant vitamins, fatty acid composition and lipid quality indices under tropical production conditions remain limited. Tropical production systems are characterised by marked seasonal variation in forage availability and quality, which can influence the supply of antioxidants and fatty acids to ruminants (Hampel et al. 2021; Ponnampalam et al. 2024a). Tropical grasses and legumes have been reported as important sources of natural antioxidants, particularly α‐tocopherol, and may contribute to improvements in lipid composition and oxidative stability of ruminant products (Hampel et al. 2021). Therefore, understanding how dietary management and genetic background are associated with hepatic lipid metabolism is essential for improving knowledge of physiological responses in tropical beef production systems and enhancing the nutritional value of bovine liver produced under these conditions.

It was hypothesised that diet‐related differences within the Nelore breed would be associated with significant changes in a broader range of hepatic lipid composition, lipid‐soluble antioxidant vitamins and lipid quality indices than breed‐associated differences observed under a common dietary regimen. It was further hypothesised that genetic background would be associated with variation in specific metabolic traits, particularly long‐chain *n‐*3 polyunsaturated fatty acids and antioxidant compounds. Therefore, the objective of this study was to evaluate breed‐associated differences under a common dietary regimen (G1 vs. G2) and diet‐related differences within the Nelore breed (G1 vs. G3) with respect to hepatic cholesterol, lipid‐soluble antioxidant vitamins, fatty acid composition and lipid quality indices. Unlike the previous study conducted with the same experimental population, which focused on the *longissimus lumborum* muscle (Conceição et al. 2026), the present study focused on liver tissue, a metabolically active organ with a central role in lipid metabolism.

## 2 Materials and Methods

### 2.1 Experimental Design, Animals and Ethical Approval

All experimental procedures were approved by the Ethics Committee for the Use of Animals of the Federal Rural University of Amazônia (UFRA; protocol no. 6613120525, ID 000837) and conducted in accordance with the ARRIVE guidelines and Directive 2010/63/EU on the protection of animals used for scientific purposes.

The experiment was carried out between July and October 2024 at the Beef Cattle Nutrition Centre (CINPEC; Integral Mix), located in Paragominas, Pará, Brazil (02°59′45″ S, 47°21′10″ W). The region has a tropical Aw climate according to the Köppen–Geiger classification, with an annual mean temperature of 26.3°C, a relative humidity of 81%, and an annual rainfall of 1742.9 mm (Bastos et al. 2006).

The study utilised liver samples from 36 feedlot‐finished bulls, comprising 24 Nelore and 12 crossbreds (½ Nelore × ½ Angus). The cattle were approximately 18 months old, with an average body weight of 430 kg and a body condition score of 3.5. Animals were allocated into three experimental groups (*n* = 12/group): G1, Nelore cattle fed a diet containing 74% concentrate and 26% *Panicum maximum* silage; G2, crossbred cattle fed the same silage–concentrate diet; and G3, Nelore cattle fed a high‐grain diet containing 85% whole maize and 15% pelleted concentrate (Table 1). The experimental design allowed comparisons of breed‐associated differences under a common dietary regimen (G1 vs. G2) and diet‐related differences within the Nelore breed (G1 vs. G3).

**Table 1:** Ingredient composition, chemical composition, antioxidant compounds and fatty acid profile of the experimental diets.

Item	Treatments		
	G1	G2	G3
Ingredient composition (% DM)			
*Panicum maximum* silage	26	26	—
Concentrate	74	74	15
Whole maize	—	—	85
Proximate composition (% as dry matter)			
Dry matter	18.3	17.3	43.4
Organicmatter	81.7	82.6	56.6
Crude protein	12.7	13.0	14.8
Neutral detergent fibre	47.2	38.6	25.1
Acid Detergent Fibre	25.7	27.6	4.92
Ash	8.61	9.68	5.38
Crude fat	4.10	3.40	4.55
Crude energy (kcal/g)	4197	4185	4290
Lipid‐soluble antioxidant compounds (µg/100 g)			
β ‐Carotene	16.6	14.7	40.4
α‐Tocopherol	64.1	83.9	67.7
α‐Tocotrienol	79.9	71.5	562
β‐Tocopherol	25.7	21.7	70.9
γ‐Tocopherol + β‐Tocotrienol	37.7	30.2	22.4
γ‐Tocotrienol	129	114	825
Δ‐Tocopherol	14.7	10.6	n.d.
Δ‐Tocotrienol	1.49	1.79	6.58
Fatty acid composition (% total fatty acid)			
14:0	0.802	0.515	0.154
15:0	0.452	0.202	0.132
16:0	20.8	19.6	16.7
18:0	3.56	0.30	0.37
18:1c9	31.0	31.6	25.3
18:2*n‐*6	31.3	30.9	43.4
18:3*n‐*3	2.71	3.08	1.65
20:0	0.730	0.650	0.496

*Note:* G1: Nelore cattle fed 74% concentrate and 26% *Panicum maximum* (Mombaça grass) silage; G2: Crossbred (½ Nelore × ½ Angus) cattle fed the same diet as G1; G3: Nelore cattle fed 85% whole maize and 15% pelleted concentrate. Diet composition was adapted from Conceição et al. (2026).

The animals used in the present study were part of the same experimental population previously described by Conceição et al. (2026). Unlike the previous study, which focused on the *longissimus lumborum* muscle, the present work evaluated liver tissue.

### 2.2 Diets, Feed Sampling and Chemical Composition

Diet samples were collected directly from the feed bunks of each experimental group at the time of feed delivery. Approximately 1 kg of feed was collected from each group and identified according to the corresponding treatment.

Samples were dried in a forced‐air oven (55°C–60°C) for 72 h, ground through a 1‐mm screen and prepared for analysis at the Animal Nutrition Laboratory (LABNUTAN), Federal Rural University of Amazônia (UFRA), Brazil.

Chemical analyses were performed at the Food Technology Laboratory of the Faculty of Veterinary Medicine and at the Animal Nutrition Laboratory of the University of Lisbon, Portugal. Dry matter, ash, crude protein, neutral detergent fibre and acid detergent fibre were determined according to the methodologies proposed by the Instituto Nacional de Ciência e Tecnologia em Ciência Animal (INCT‐CA) (Detmann et al. 2012). Non‐fibrous carbohydrates were determined according to Sniffen et al. (1992), and total digestible nutrients were estimated using the Clemson University equation. The ingredient composition, chemical composition, lipid‐soluble antioxidant compounds and fatty acid profile of the experimental diets are presented in Table 1.

Although G1 and G2 received the same dietary formulation, feed samples were collected independently from the feed bunks of each group and analysed separately. Consequently, the small differences observed in the chemical composition presented in Table 1 reflect normal sampling and analytical variation among feed samples rather than intentional differences in the diet offered to the animals.

### 2.3 Slaughter Procedures and Liver Sampling

After a 14‐h fasting period, animals were weighed and transported to a commercial abattoir, where slaughter procedures were performed in accordance with the Brazilian Regulation for Industrial and Sanitary Inspection of Products of Animal Origin (RIISPOA; Brazil 2020).

Liver samples (approximately 210 g) were collected 24 h *post‐mortem*, vacuum‐packed and stored at −80°C until analysis.

### 2.4 Liver Sample Preparation

Samples were subsequently freeze‐dried using a laboratory freeze dryer (L101, Liobras, São Carlos, SP, Brazil) with a working temperature of up to −55°C. During lyophilisation, samples were maintained under vacuum (158–256 mmHg) at condenser temperatures of −42°C to −45°C for approximately 48 h, thereby allowing water removal by sublimation prior to chemical analyses.

### 2.5 Determination of Cholesterol and Lipid‐Soluble Antioxidant Compounds

Hepatic cholesterol, β‐carotene and vitamin E homologues (tocopherols and tocotrienols) were determined as described by Prates (2006). Analyses were performed by high‐performance liquid chromatography (HPLC; Agilent 1100, Agilent Technologies, Santa Clara, CA, USA) using a normal‐phase silica column (Zorbax Rx‐Sil, 5 μm, 4.6 × 250 mm) maintained at 20°C. The mobile phase consisted of *n‐*hexane:2‐propanol (99:1, v/v) at a flow rate of 1.0 mL/min.

Chromatographic detection was performed using fluorescence (FLD) and UV–Vis detectors. Tocopherols and tocotrienols were detected by fluorescence at excitation 295 nm and emission 325 nm. Cholesterol and β‐carotene were detected by UV–Vis at 202 and 450 nm, respectively.

Quantification was performed using the external standard method based on calibration curves. Cholesterol was quantified using Cholesterol Sigma Grade (≥ 99%; Sigma‐Aldrich, St. Louis, MO, USA), β‐carotene using a European Pharmacopoeia (EP) Reference Standard, and tocopherol homologues using a Tocopherol Set containing all‐rac‐α‐tocopherol, all‐rac‐β‐tocopherol, RRR‐γ‐tocopherol and RRR‐δ‐tocopherol standards (Merck/Sigma‐Aldrich, Darmstadt, Germany). Calibration curves were constructed by plotting peak area against standard concentration for each analyte.

### 2.6 Lipid Extraction and Fatty Acid Analysis

Total lipids were extracted from freeze‐dried liver samples according to Folch et al. (1957), as modified by Carlson (1985), and quantified gravimetrically. Fatty acid methyl esters (FAME) were prepared according to Pestana (2020) and analysed by gas chromatography with flame ionisation detection (GC‐FID; HP 7890A, Hewlett‐Packard, USA). Separation was achieved using a Supelcowax 10 capillary column (100 m × 0.20 mm × 0.20 μm; Supelco, Bellefonte, PA, USA) under chromatographic conditions described by Alves and Bessa (2009). Injector and detector temperatures were set at 250°C and 280°C, respectively. Helium was used as the carrier gas at a flow rate of 1.0 mL/min. Samples were injected in split mode (1:50) with an injection volume of 1 μL. Heneicosanoic acid methyl ester (21:0) was used as an internal standard. Fatty acids were identified using a commercial 37‐component FAME standard mixture (Supelco Inc., USA) and expressed as g/100 g total fatty acids.

### 2.7 Lipid Nutritional Quality Indices

The fatty acid profile of liver samples was expressed as a percentage of total identified fatty acids. From these data, nutritional quality indices were calculated to estimate the potential effects of hepatic lipids on human health.

The PUFA/SFA and *n‐*6/*n‐*3 ratios were obtained from the sums of polyunsaturated, saturated, *n‐*6 and *n‐*3 fatty acids, respectively. Hypocholesterolaemic (h) and hypercholesterolaemic (H) fatty acid fractions were determined following Ulbricht and Southgate (1991), and the h/H ratio was subsequently calculated. The atherogenicity (IA) and thrombogenicity (IT) indices were also estimated using the equations proposed by Ulbricht and Southgate (1991). Lower IA and IT values are associated with reduced atherogenic and thrombogenic potential of dietary lipids.

These indices were used as integrated indicators of hepatic lipid nutritional quality and cardiovascular health‐related properties.

### 2.8 Statistical Analysis

Because the experimental design consisted of three predefined experimental groups and did not include all breed × diet combinations, the data were analysed as a comparative study among groups rather than as a factorial experiment (Figure 1). Each animal was considered the experimental unit. Data were analysed in R (R Core Team).

**Figure 1:**
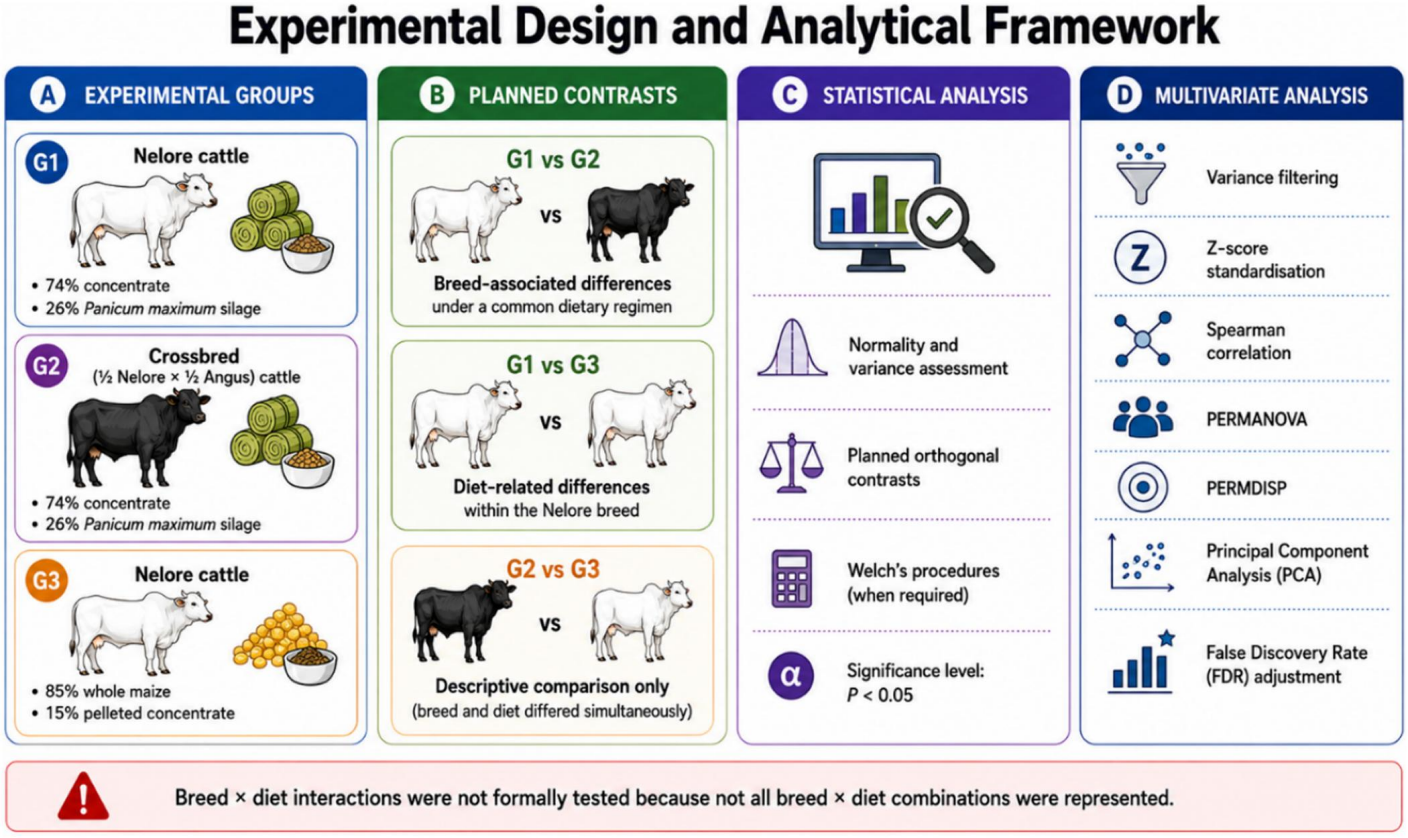
Experimental design and analytical framework. Schematic representation of the experimental groups, planned contrasts and statistical procedures used in the study. Breed‐associated differences were evaluated by the G1 versus G2 contrast, whereas diet‐related differences within the Nelore breed were evaluated by the G1 versus G3 contrast. The G2 versus G3 comparison was descriptive only. [Color figure can be viewed at wileyonlinelibrary.com]

Planned orthogonal contrasts were used to evaluate breed‐associated differences under a common dietary regimen (G1 vs. G2) and diet‐related differences within the Nelore breed (G1 vs. G3). An additional comparison between G2 and G3 was reported for descriptive purposes only because these groups differed simultaneously in breed and diet. As not all breed × diet combinations were represented, breed × diet interactions could not be formally tested.

Residual normality and homogeneity of variances were assessed using the Shapiro–Wilk and Bartlett tests, respectively. When heteroscedasticity was detected, Welch's ANOVA followed by Welch's *t*‐test was applied.

Data are presented as means ± SEM. Differences were considered significant at *p* < 0.05, whereas values between 0.05 and 0.10 were considered tendencies.

Multivariate analyses were performed after variance filtering and data standardisation. Differences among groups were evaluated using PERMANOVA, and homogeneity of multivariate dispersions was assessed using PERMDISP. Principal component analysis (PCA) was used to visualise sample distribution patterns, and pairwise comparisons were adjusted using the false discovery rate (FDR) method.

## 3 Results

### 3.1 Cholesterol and Lipid‐Soluble Antioxidant Vitamins

Liver cholesterol and lipid‐soluble antioxidant vitamin concentrations are presented in Table 2. No significant differences in liver cholesterol concentration were observed among the evaluated contrasts (*p* > 0.05).

**Table 2:** Cholesterol (mg/g) and lipid‐soluble antioxidant vitamins (µg/g) in liver (values are mean ± SEM; *n* = 12).

Variable	G1	G2	G3	*p‐*value (Contrast)			*p*‐value
				Breed (G1 vs. G2)	Diet (G1 vs. G3)	Breed/Diet (G2 vs. G3)	
Cholesterol	2.34 ± 0.064	2.50 ± 0.133	2.54 ± 0.259	0.516	0.867	0.416	0.687
β‐Carotene	9.82 ± 3.451	25.47 ± 5.889	2.77 ± 0.646	0.008	< 0.001	0.216	0.001
α‐Tocopherol	14.29 ± 1.829	18.56 ± 1.997	6.30 ± 0.681	0.070	< 0.001	0.001	< 0.001
β‐Tocopherol	0.34 ± 0.106	0.66 ± 0.101	0.19 ± 0.025	0.012	< 0.001	< 0.226	0.001
γ‐Tocopherol	0.08 ± 0.020	0.04 ± 0.004	0.04 ± 0.006	0.028	0.975	0.026	0.040
Δ‐Tocopherol	0.06 ± 0.002	0.07 ± 0.003	0.05 ± 0.004	0.306	0.002	0.027	0.006
α‐Tocotrienol	0.17 ± 0.012	0.15 ± 0.014	0.06 ± 0.011	0.299	< 0.001	< 0.001	< 0.001
γ‐Tocotrienol	0.05 ± 0.007	0.06 ± 0.005	0.11 ± 0.013	0.461	< 0.001	< 0.001	< 0.001

*Note:* G1: Nelore cattle fed 74% concentrate and 26% *Panicum maximum* (Mombaça grass) silage; G2: Crossbred (½ Nelore × ½ Angus) cattle fed the same diet as G1; G3: Nelore cattle fed 85% whole maize and 15% pelleted concentrate. G2 versus G3 (Breed/Diet) represents a descriptive comparison only.

Abbreviation: SEM, standard error of the mean.

Breed‐associated differences were detected for β‐carotene, β‐tocopherol and γ‐tocopherol (all *p* < 0.05), whereas α‐tocopherol showed a tendency towards significance (*p* = 0.070). Diet‐related differences were observed for β‐carotene, α‐tocopherol, β‐tocopherol, Δ‐tocopherol, α‐tocotrienol and γ‐tocotrienol (all *p* ≤ 0.002).

Consistent with these results, principal component analysis (Figure 2) showed a clear separation of G3 from G1 and G2 along Dim1 (43% of the total variance), while G1 and G2 exhibited partial overlap. β‐Carotene, α‐tocopherol, α‐tocotrienol and γ‐tocotrienol were the variables most strongly associated with sample distribution in the multivariate space.

**Figure 2:**
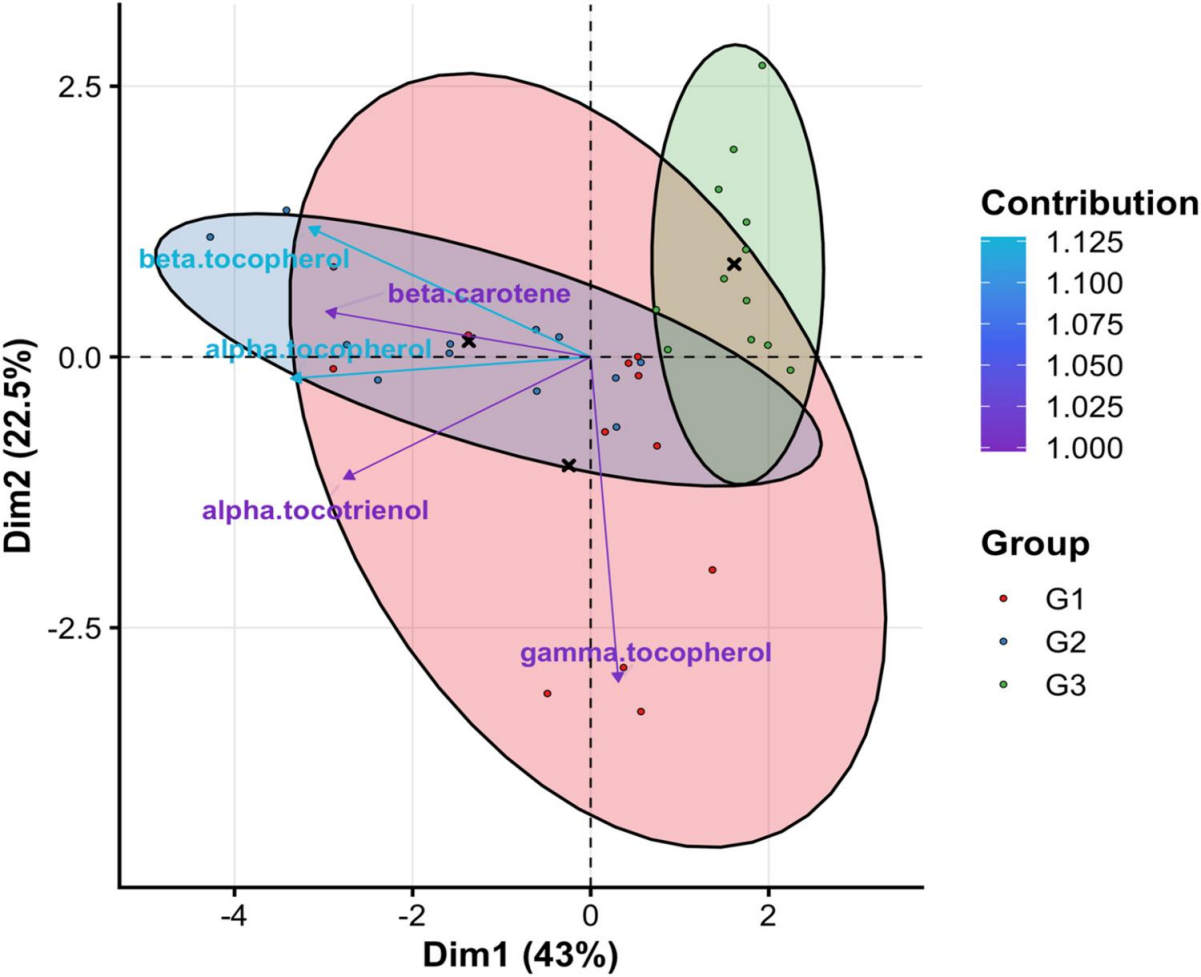
Principal component analysis (PCA) of cholesterol and lipid‐soluble antioxidant vitamins in cattle liver. The first two principal components explained 68% of the total variance (Dim1 = 43% and Dim2 = 22.5%). Points represent individual animals, and ellipses indicate group clustering. Vectors show the contribution and direction of associations between cholesterol, lipid‐soluble antioxidant vitamins and the sample distribution. G1: Nelore cattle fed 74% concentrate and 26% Panicum maximum (Mombaça grass) silage; G2: crossbred (½ Nelore × ½ Angus) cattle fed the same diet as G1; G3: Nelore cattle fed 85% whole maize and 15% pelleted concentrate. [Color figure can be viewed at wileyonlinelibrary.com]

### 3.2 Total Lipid and Fatty Acid Profile

Total lipid content and fatty acid composition in the liver are presented in Table 3. The contrast representing breed‐associated differences (G1 vs. G2) was significant only for docosahexaenoic acid (22:6*n‐*3; *p* = 0.012). No breed‐associated differences were detected for total lipid content or for the remaining fatty acids (*p* > 0.05).

**Table 3:** Total lipid (mg/g) and fatty acid composition (% total fatty acid) in livers (values are mean ± SEM; *n* = 12).

Fatty acid	G1	G2	G3	*p‐*value (Contrast)			*p‐*value
				Breed (G1 vs. G2)	Diet (G1 vs. G3)	Breed/Diet (G2 vs. G3)	
Total lipid	3.42 ± 0.258	3.12 ± 0.222	2.56 ± 0.195	0.354	0.092	0.011	0.036
12:0	0.05 ± 0.005	0.04 ± 0.010	0.12 ± 0.022	0.623	< 0.001	0.001	< 0.001
14:0	0.16 ± 0.020	0.20 ± 0.031	0.21 ± 0.012	0.186	0.765	0.108	0.228
15:0	0.12 ± 0.006	0.11 ± 0.007	0.20 ± 0.016	0.982	< 0.001	< 0.001	< 0.001
16:0	9.94 ± 0.365	9.80 ± 0.365	8.42 ± 0.366	0.802	0.019	0.010	0.019
16:1 9c	0.47 ± 0.047	0.53 ± 0.037	0.47 ± 0.038	0.267	0.291	0.954	0.454
17:00	0.55 ± 0.023	0.56 ± 0.019	1.07 ± 0.096	0.922	< 0.001	< 0.001	< 0.001
17:1 9c	0.14 ± 0.017	0.17 ± 0.013	0.31 ± 0.037	0.398	< 0.001	< 0.001	< 0.001
18:0	34.42 ± 0.502	34.97 ± 0.692	36.30 ± 1.019	0.617	0.227	0.092	0.219
18:1 9c	10.57 ± 0.574	11.78 ± 0.474	8.67 ± 0.508	0.110	< 0.001	0.014	< 0.001
Ʃ18 cis	2.91 ± 0.340	3.05 ± 0.377	4.46 ± 0.636	0.825	0.042	0.025	0.048
18:1 t10	0.37 ± 0.036	0.38 ± 0.019	0.92 ± 0.175	0.928	0.001	< 0.001	< 0.001
Ʃ18 trans	0.76 ± 0.142	0.51 ± 0.038	0.70 ± 0.097	0.092	0.184	0.708	0.205
CLA	0.28 ± 0.020	0.29 ± 0.019	0.28 ± 0.021	0.680	0.672	0.990	0.889
18:2*n‐*6	19.81 ± 0.589	19.39 ± 0.555	21.10 ± 0.978	0.684	0.107	0.222	0.242
18:3*n‐*6	0.10 ± 0.009	0.11 ± 0.017	0.11 ± 0.017	0.719	0.892	0.719	0.935
18:3*n‐*3	1.15 ± 0.055	1.03 ± 0.053	0.70 ± 0.036	0.101	< 0.001	< 0.001	< 0.001
20:0	0.09 ± 0.004	0.09 ± 0.003	0.12 ± 0.009	0.953	0.017	0.020	0.026
20:2*n‐*6	0.42 ± 0.021	0.37 ± 0.022	0.60 ± 0.042	0.259	< 0.001	< 0.001	< 0.001
20:3*n‐*6	0.22 ± 0.011	0.22 ± 0.012	0.24 ± 0.014	0.953	0.363	0.333	0.552
20:4*n‐*6	7.91 ± 0.276	8.01 ± 0.291	7.31 ± 0.467	0.843	0.169	0.236	0.327
22:0	3.72 ± 0.360	2.97 ± 0.340	2.55 ± 0.348	0.140	0.402	0.024	0.071
22:5*n‐*3	2.20 ± 0.076	2.21 ± 0.120	1.72 ± 0.125	0.968	0.003	0.004	0.004
22:6*n‐*3	1.97 ± 0.076	1.45 ± 0.120	1.77 ± 0.191	0.012	0.103	0.331	0.038
23:0	0.10 ± 0.007	0.11 ± 0.010	0.14 ± 0.014	0.523	0.131	0.035	0.094
24:0	0.48 ± 0.023	0.41 ± 0.018	0.34 ± 0.030	0.055	0.048	< 0.001	0.001
Non‐identified	0.97 ± 0.103	1.09 ± 0.081	1.07 ± 0.213	0.543	0.916	0.615	0.807

*Note:* G1: Nelore cattle fed 74% concentrate and 26% *Panicum maximum* (Mombaça grass) silage; G2: Crossbred (½ Nelore × ½ Angus) cattle fed the same diet as G1; G3: Nelore cattle fed 85% whole maize and 15% pelleted concentrate. G2 versus G3 (Breed/Diet) represents a descriptive comparison only.

Abbreviation: SEM, standard error of the mean.

The contrast representing diet‐related differences (G1 vs. G3) significantly affected 13 fatty acids, including saturated, monounsaturated and polyunsaturated fatty acids (all *p* < 0.05). Significant differences were observed for 18:3*n‐*3 and 22:5*n‐*3.

Overall, significant differences were detected for a larger number of fatty acids in the diet‐related contrast (G1 vs. G3) than in the breed‐associated contrast (G1 vs. G2).

The partial sums of fatty acids in the liver are presented in Table 4. The contrast representing breed‐associated differences (G1 vs. G2) was significant only for total *n‐*3 polyunsaturated fatty acids (Σ*n‐*3 PUFA; *p* = 0.022). The contrast representing diet‐related differences (G1 vs. G3) was significant for total monounsaturated fatty acids (ΣMUFA; *p* < 0.001), whereas Σ*n‐*3 PUFA showed a tendency towards significance (*p* = 0.068).

**Table 4:** Partial sums of liver fatty acids (values are mean ± SEM).

					*p‐*value (Contrast)		*p‐*value
Item	G1	G2	G3	Breed (G1 vs. G2)	Diet (G1 vs. G3)	Breed/Diet (G2 vs. G3)	
ƩSFA	49.67 ± 0.363	49.67 ± 0.362	49.50 ± 0.081	0.674	0.820	0.846	0.913
ƩMUFA	11.19 ± 0.624	12.49 ± 0.507	9.45 ± 0.543	0.109	< 0.001	0.036	0.002
ƩPUFA	14.01 ± 0.344	13.44 ± 0.346	12.47 ± 0.740	0.436	0.189	0.041	0.115
Ʃ*n‐*3 PUFA	5.33 ± 0.118	4.70 ± 0.113	4.20 ± 0.275	0.022	0.068	< 0.001	< 0.001
Ʃ*n‐*6 PUFA	28.49 ± 0.734	28.13 ± 0.540	29.37 ± 1.226	0.770	0.327	0.488	0.598

*Note:* G1: Nelore cattle fed 74% concentrate and 26% *Panicum maximum* (Mombaça grass) silage; G2: Crossbred (½ Nelore × ½ Angus) cattle fed the same diet as G1; G3: Nelore cattle fed 85% whole maize and 15% pelleted concentrate. G2 versus G3 (Breed/Diet) represents a descriptive comparison only.

Abbreviation: SEM, standard error of the mean.

Multivariate analysis of hepatic fatty acid composition revealed significant differences among experimental groups (PERMANOVA, *p* = 0.003, R^2^ = 0.222). Group dispersions did not differ significantly (PERMDISP, *p* = 0.174), indicating that the observed multivariate differences were not attributable to differences in within‐group dispersion.

Principal component analysis explained 50.3% of the total variance in the first two components (PC1 = 33.6%; PC2 = 16.7%), showing partial separation among the experimental groups (Figure 3).

**Figure 3:**
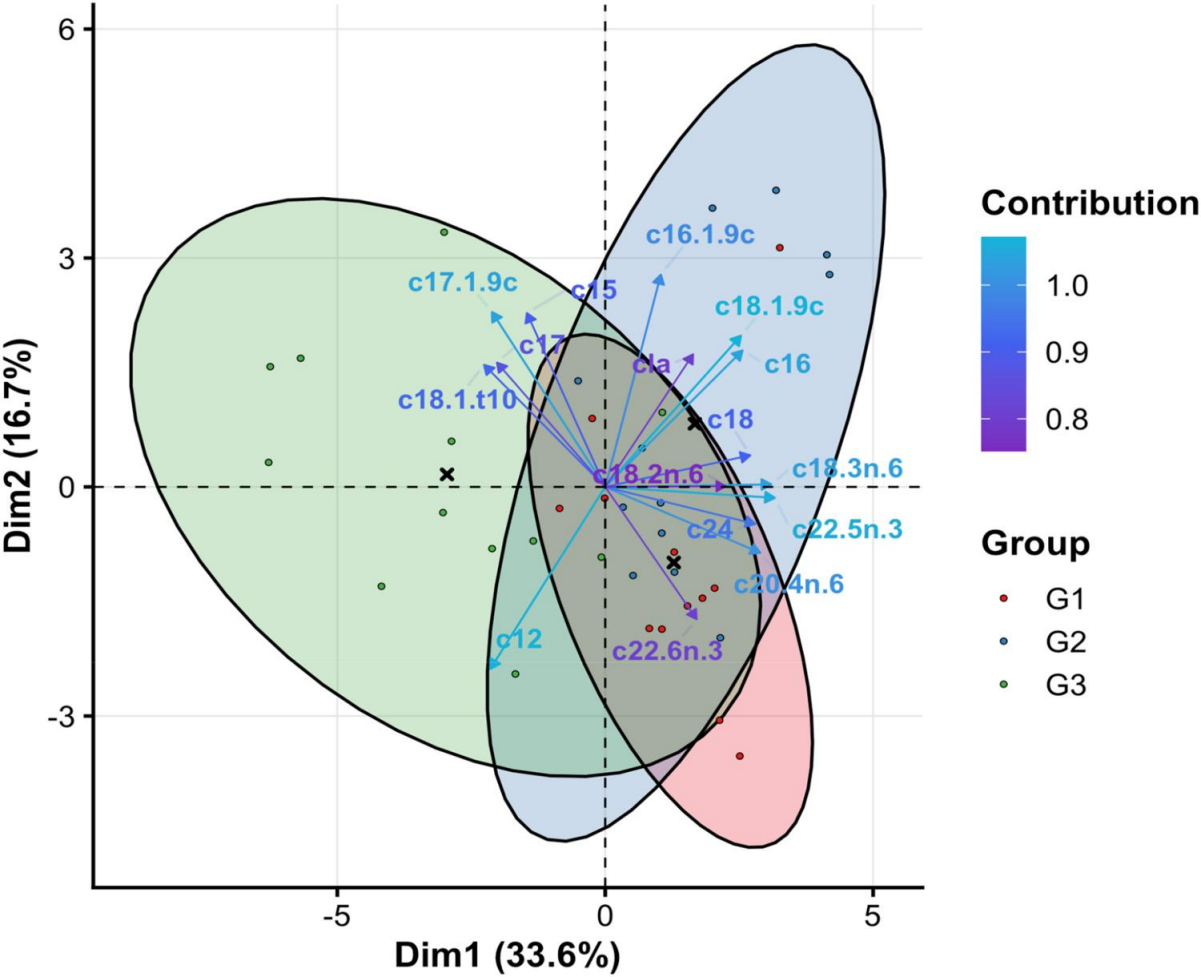
Principal component analysis (PCA) biplot of hepatic fatty acid composition in beef cattle. The first two principal components explained 50.3% of the total variance (PC1 = 33.6%; PC2 = 16.7%). Ellipses represent the 95% confidence intervals for each experimental group. G1: Nelore cattle fed a silage–concentrate diet; G2: crossbred (½ Nelore × ½ Angus) cattle fed the same silage–concentrate diet; G3: Nelore cattle fed a high‐grain diet. Vectors indicate the fatty acids contributing to sample discrimination. [Color figure can be viewed at wileyonlinelibrary.com]

The multivariate separation was primarily associated with variation in long‐chain polyunsaturated fatty acids, particularly C18:2*n‐*6, C20:4*n‐*6, C22:5*n‐*3 and C18:3*n‐*3.

Pairwise multivariate comparisons showed no significant difference between G1 and G2 (FDR‐adjusted *p* = 0.210), whereas G1 and G3 differed significantly (FDR‐adjusted *p* = 0.002). Accordingly, multivariate differences in hepatic fatty acid composition were detected for the diet‐related contrast within the Nelore breed but not for the breed‐associated contrast evaluated under a common dietary regimen.

### 3.3 Nutritional Indices of Fatty Acids

Nutritional ratios and lipid quality indices in the liver are presented in Table 5. No breed‐associated differences were detected for any of the evaluated indices (*p* > 0.05). Diet‐related differences were observed for the *n‐*6/*n‐*3 ratio, hypocholesterolaemic index (h) and hypercholesterolaemic index (H) (all *p* < 0.05), whereas the thrombogenicity index (IT) showed a tendency towards significance (*p* = 0.067).

**Table 5:** Nutritional rations and indices of liver (values are mean ± SEM).

Item	G1	G2	G3	*p*‐value (Contrast)			*p*‐value
				Breed contrast (G1 vs. G2)	Diet contrast (G1 vs. G3)	Breed/Diet (G2 vs. G3)	
PUFA/SFA	0.28 ± 0.006	0.27 ± 0.006	0.25 ± 0.017	0.554	0.269	0.094	0.232
*n‐*6/*n‐*3	5.37 ± 0.185	6.01 ± 0.165	7.22 ± 0.397	0.102	0.003	< 0.001	< 0.001
h	44.29 ± 0.513	44.50 ± 0.594	42.14 ± 1.113	0.854	0.041	0.061	0.078
H	10.16 ± 0.375	10.05 ± 0.472	8.76 ± 0.378	0.854	0.033	0.022	0.039
h/H	4.444 ± 0.219	4.53 ± 0.219	4.97 ± 0.374	0.820	0.280	0.193	0.376
IA	0.23 ± 0.010	0.23 ± 0.013	0.22 ± 0.013	0.951	0.414	0.380	0.615
IT	1.23 ± 0.016	1.30 ± 0.014	1.42 ± 0.073	0.315	0.067	0.006	0.020

*Note:* G1: Nelore cattle fed 74% concentrate and 26% *Panicum maximum* (Mombaça grass) silage; G2: Crossbred (½ Nelore × ½ Angus) cattle fed the same diet as G1; G3: Nelore cattle fed 85% whole maize and 15% pelleted concentrate. G2 versus G3 (Breed/Diet) represents a descriptive comparison only.

Abbreviation: SEM, standard error of the mean.

## 4 Discussion

The present study evaluated breed‐associated differences under a common dietary regimen and diet‐related differences within the Nelore breed. Overall, significant differences were detected for a greater number of hepatic lipid and antioxidant variables in the diet‐related contrast (G1 vs. G3) than in the breed‐associated contrast (G1 vs. G2). The stability of hepatic cholesterol, together with the dietary‐related changes observed for carotenoids, tocopherols and fatty acids, highlights the liver's central role in regulating lipid homoeostasis and responding to nutritional variation.

These findings align with current evidence that the liver functions as both a metabolic integrator and a sensitive biomarker of nutritional status in ruminants (Lobato et al. 2024; Ponnampalam et al. 2024a). The responses observed in the present study provide additional insights into how feeding strategies in tropical beef systems influence hepatic composition, with implications for both animal performance and human nutrition.

Hepatic cholesterol concentration remained remarkably stable across breed‐associated and diet‐related differences, suggesting that hepatic cholesterol homeostasis is tightly regulated despite nutritional and genetic influences. This finding is consistent with Lobato et al. (2024), who reported no significant variation among production systems, and with Prates (2006), whose values for extensively reared Barrosã cattle were within the range observed herein. In contrast, Rodrigues et al. (2023) reported significant effects of interactions between ecosystem and season on hepatic cholesterol concentration in buffaloes, suggesting that environmental and management conditions may influence this metabolite. Therefore, the breed‐associated and diet‐related differences evaluated in the present study were insufficient to alter hepatic cholesterol concentration.

Lipid‐soluble antioxidant vitamins were among the most responsive hepatic components to dietary and genetic variation, indicating that antioxidant deposition is highly sensitive to nutritional management and, to a lesser extent, genetic background. The higher concentrations of β‐carotene, α‐tocopherol and β‐tocopherol observed in specific groups are consistent with Schulz et al. (2025), who reported increased hepatic β‐carotene concentrations in animals with greater pasture availability. Similarly, Álvarez et al. (2015) and Nozière et al. (2006) demonstrated that carotenoid and vitamin deposition in ruminant tissues closely reflects dietary composition and forage intake. Block and Farmer (1987) additionally reported increased plasma and hepatic β‐carotene and vitamin A concentrations in animals receiving higher forage proportions. Collectively, these findings suggest that hepatic antioxidant deposition is highly responsive to dietary composition and may serve as a useful indicator of nutritional management in feedlot cattle.

The marked response of tocopherols to dietary variation further supports the sensitivity of hepatic antioxidant metabolism to nutritional management. This finding is consistent with Lobato et al. (2024), Liu et al. (1995) and Descalzo and Sancho (2008), who demonstrated that nutritional management and forage‐based systems improve antioxidant deposition and oxidative stability in bovine tissues. Rodrigues et al. (2023) further reported significant effects of ecosystem and season on hepatic α‐tocopherol and γ‐tocopherol concentrations, reinforcing the influence of forage quality and availability on antioxidant status. Together, these findings indicate that tocopherol deposition in the liver is strongly dependent on dietary antioxidant supply. This interpretation is further supported by Hampel et al. (2021), who reported that tropical pastures contain high concentrations of natural antioxidants, particularly α‐tocopherol, resulting in tissue concentrations associated with improved oxidative stability of ruminant products. Although conducted in lambs, these findings reinforce the importance of forage‐derived antioxidants as determinants of tissue vitamin E deposition in tropical production systems.

The significant breed‐associated differences observed for β‐carotene, β‐tocopherol and γ‐tocopherol suggest that the metabolism and deposition of lipid‐soluble antioxidants are partially influenced by genetic background. Serviço Nacional de Aprendizagem Rural SENAR (2018) described differences between taurine and zebu cattle in nutrient utilisation efficiency and fat deposition, whereas Zachwieja et al. (2025) reported breed‐associated variation in polyunsaturated fatty acid deposition, including 18:3*n‐*3 and 22:6*n‐*3.

The clear separation of G3 from G1 and G2 observed in the PCA indicates that dietary composition produced distinct hepatic antioxidant profiles, whereas breed‐associated differences generated comparatively smaller metabolic shifts. Krusinski et al. (2024) similarly identified α‐tocopherol and the *n‐*6:*n‐*3 ratio as important discriminating variables among dietary groups, whereas Makhutova et al. (2023) demonstrated the usefulness of PCA for describing variation in hepatic lipid metabolism. Together, these findings reinforce the usefulness of hepatic antioxidant compounds as biomarkers of nutritional management and metabolic adaptation in beef cattle.

The fatty acid profile showed that diet‐related differences within the Nelore breed were associated with significant changes in more fatty acids than breed‐associated differences evaluated under a common dietary regimen. In contrast, docosahexaenoic acid (22:6*n‐*3) was the only fatty acid significantly affected by the breed‐associated contrast. These findings indicate that significant differences in hepatic fatty acid composition were more frequently detected for the diet‐related contrast within the Nelore breed, whereas breed‐associated differences under a common dietary regimen were restricted mainly to specific long‐chain *n‐*3 PUFA.

The greater number of significant diet‐related differences observed in the present study is consistent with Alfaia et al. (2009) and De la Fuente et al. 2009), who demonstrated that dietary composition and production system are the principal determinants of fatty acid composition in ruminant tissues. Costa et al. (2014) observed that the dietary silage level significantly affected hepatic 18:3*n‐*3, 22:5*n‐*3 and 22:6*n‐*3 concentrations, whereas Xu et al. (2015) demonstrated that high‐concentrate diets markedly altered hepatic fatty acid metabolism. Similarly, Ponnampalam et al. (2024a, 2024b) reported that forage‐based systems favour *n‐*3 polyunsaturated fatty acid deposition, whereas grain‐based diets increase *n‐*6 fatty acids and the *n‐*6:*n‐*3 ratio.

Despite the predominance of dietary effects, the significant breed‐associated difference observed for 22:6*n‐*3 suggests that genetic background contributes to the regulation of long‐chain *n‐*3 polyunsaturated fatty acid metabolism. Similar results were reported by Costa et al. (2014) and Zachwieja et al. (2025), who observed breed‐related variation in hepatic DHA deposition, reinforcing the role of genotype in specific aspects of lipid metabolism.

Overall, these findings indicate that diet‐related differences within the Nelore breed were associated with broader changes in hepatic fatty acid composition, whereas breed‐associated differences were limited to fewer variables.

Nutritional lipid quality indices were affected by the diet‐related contrast, whereas no significant breed‐associated differences were detected for any of the evaluated indices. These findings suggest that genetic background contributes little to the overall nutritional quality of hepatic lipids under the conditions evaluated. Li et al. (2014) similarly observed no significant differences in the atherogenicity index (IA) between purebred and crossbred cattle, whereas Costa et al. (2014) reported relative stability of lipid‐related parameters between breeds.

Significant diet‐related differences were observed for the *n‐*6:*n‐*3 ratio and for the hypocholesterolaemic and hypercholesterolaemic indices. Rodrigues et al. (2023) demonstrated marked effects of production system on hepatic *n‐*6:*n‐*3 ratio and h/H fractions in buffalo liver, whereas Silva et al. (2022) reported more favourable lipid quality indices in pasture‐raised animals. Similarly, Alfaia et al. (2009) and Ponnampalam et al. (2024a, 2024b) demonstrated that forage‐based systems favour greater deposition of *n‐*3 fatty acids and more favourable lipid quality indices for human consumption. Supporting this interpretation, Hampel et al. (2021) reported *n‐*6/*n*3 ratios below 2:1 in lambs raised on tropical grass pastures, highlighting the capacity of tropical forage systems to improve the nutritional quality of ruminant‐derived foods through enhanced *n‐*3 fatty acid deposition. These findings indicate that the diet‐related contrast affected several hepatic lipid quality indices, whereas no significant effects were detected for the breed‐associated contrast.

Overall, the present findings indicate that the diet‐related contrast within the Nelore breed was associated with significant changes in hepatic antioxidant status, fatty acid composition and lipid quality indices, whereas the breed‐associated contrast under a common dietary regimen affected a more limited number of variables, particularly those related to long‐chain *n‐*3 polyunsaturated fatty acids.

## 5 Conclusion

The results indicate that significant diet‐related differences within the Nelore breed were observed for more hepatic lipid, lipid‐soluble antioxidant and lipid quality variables than breed‐associated differences evaluated under a common dietary regimen. Genetic background exerted a more limited effect, mainly influencing the deposition of specific long‐chain polyunsaturated fatty acids and lipid‐soluble antioxidants.

The feeding strategies evaluated significantly altered hepatic fatty acid composition, antioxidant vitamin concentrations and nutritional lipid quality indices, reinforcing the central role of diet in modulating hepatic metabolism in beef cattle. Multivariate analyses further demonstrated that lipid‐soluble antioxidant compounds are sensitive biomarkers of metabolic and nutritional differences among the experimental groups.

However, the study was conducted under specific management conditions and with a limited number of genetic groups, which may limit the generalisability of the findings to other production systems and environmental conditions. Furthermore, the molecular mechanisms involved in the regulation of hepatic lipid metabolism were not directly evaluated.

Future studies should investigate the nutrigenomic mechanisms underlying hepatic metabolism, including genes involved in lipid transport, oxidation and deposition, and should evaluate different production systems, genetic groups and environmental conditions to improve understanding of how diet and genetic background interact to determine the nutritional quality of bovine liver.

## Author Contributions

**Laíza de Kássia Mendes da Conceição:** writing – original draft, investigation, methodology. **José de Brito Lourenço Júnior:** writing – review and editing, validation and supervision. **Jamile Andréa Rodrigues da Silva:** writing – review and editing, validation and supervision. **Leonel António Joaquim:** data curation, methodology. **Mariana Jucá Moraes:** conceptualization. **Thomaz Cyro Guimarães de Carvalho Rodrigues:** original draft, methodology. **Joana Inês Ferreira:** methodology, investigation. **Ana Maria Ramos Fontes:** methodology. **Maria Pinheiro Spínola:** methodology, investigation. **Érica Coutinho David:** methodology. **Antônio Vinicius Correa Barbosa:** methodology, investigation, formal analysis. **Bruno Soares Cabral:** original draft, methodology. **José António Mestre Prates:** writing – review and editing, validation and supervision.

## Ethics Statement

All experimental procedures involving animals were conducted in strict accordance with the ethical standards and welfare policies of the Journal of Animal Physiology and Animal Nutrition. The study protocol was reviewed and approved by the Ethics Committee for the Use of Animals (CEUA) of the Federal Rural University of Amazônia (UFRA), under approval number 6613120525 (ID 000837). All procedures complied with the ARRIVE guidelines and followed the principles established in the European Directive 2010/63/EU on the protection of animals used for scientific purposes.

## Conflicts of Interest

The authors declare no conflicts of interest.

## Data Availability

The data that support the findings of this study are available from the corresponding author upon reasonable request.
